# Usability-In-Place—Remote Usability Testing Methods for Homebound Older Adults: Rapid Literature Review

**DOI:** 10.2196/26181

**Published:** 2021-11-02

**Authors:** Jordan R Hill, Janetta C Brown, Noll L Campbell, Richard J Holden

**Affiliations:** 1 Department of Pharmacy Practice College of Pharmacy Purdue University Indianapolis, IN United States; 2 Indiana University Center for Aging Research Regenstrief Institute Indianapolis, IN United States; 3 School of Medicine Indiana University Indianapolis, IN United States; 4 Center for Health Innovation and Implementation Science Indiana University Indianapolis, IN United States; 5 Department of Health and Wellness Design School of Public Health-Bloomington Indiana University Bloomington, IN United States

**Keywords:** mobile usability testing, usability inspection, methods, aging, literature synthesis, usability study, mobile usability, elderly, older adults, remote usability, mobility restriction

## Abstract

**Background:**

Technology can benefit older adults in many ways, including by facilitating remote access to services, communication, and socialization for convenience or out of necessity when individuals are homebound. As people, especially older adults, self-quarantined and sheltered in place during the COVID-19 pandemic, the importance of usability-in-place became clear. To understand the remote use of technology in an ecologically valid manner, researchers and others must be able to test usability remotely.

**Objective:**

Our objective was to review practical approaches for and findings about remote usability testing, particularly remote usability testing with older adults.

**Methods:**

We performed a rapid review of the literature and reported on available methods, their advantages and disadvantages, and practical recommendations. This review also reported recommendations for usability testing with older adults from the literature.

**Results:**

Critically, we identified a gap in the literature—a lack of remote usability testing methods, tools, and strategies for older adults, despite this population’s increased remote technology use and needs (eg, due to disability or technology experience). We summarized existing remote usability methods that were found in the literature as well as guidelines that are available for conducting in-person usability testing with older adults.

**Conclusions:**

We call on the human factors research and practice community to address this gap to better support older adults and other homebound or mobility-restricted individuals.

## Introduction

### The Need for Remote Operations

Technology can support access to services, communication, and socialization for older adults and others whose mobility is restricted due to health-related risks such as susceptibility to disease (eg, COVID-19), disability, and a lack of resources (eg, transportation). However, the delivery, support, and evaluation of technologies that are used by homebound or mobility-restricted individuals require remote operations, including remote usability testing. Herein, we review the unique needs and technology opportunities of homebound older adults and the literature on remote usability testing methods. Based on our findings, we identified a gap in guidance for remote usability testing with older adults. Therefore, we call on relevant research and practice communities to address this gap.

### Supporting Homebound Individuals With Technology

Over 2 million Americans are homebound due to an array of social, functional, and health-related causes, and this number is projected to grow as the size of the older population increases [1]. Situational factors such as inclement weather and, on a larger scale, pandemics or national disasters can also temporarily render individuals homebound. For example, in March 2020, the US Centers for Disease Control and Prevention [2] warned older adults to remain at home due to the disproportionate COVID-19–related health risks that they face. Prior to the pandemic, an estimated 1 in 4 older US adults were already socially isolated, and this rate has likely increased [3].

People who shelter in place or stay home for other reasons may turn to technology to access remote services, including remote banking, grocery shopping, and medical care services. The prevalence of these physically distant interactions is reportedly on the rise [4], especially for certain services. A prominent example is the increased frequency of patients’ telemedicine visits with health care professionals—a form of telehealth that has been long available but whose usage has increased dramatically in the United States, as the COVID-19 pandemic resulted in changes to federal reimbursement policies in March 2020 [5].

### Testing Technology With Homebound Technology Users

When technology users are homebound, researchers and care practitioners who intend to test a technology’s usability in an ecologically valid manner must either travel to the user’s home or conduct remote testing. Travel is not always an option. Safety, health, or personal reasons may prevent researchers from entering a home or community. Travel may be too costly or otherwise impractical, or participants may live in an area that is inaccessible to the project team. During the COVID-19 pandemic for example, academic and practice-based project teams have anecdotally reported barriers to in-person visits, including the need to distance themselves from infected and at-risk individuals, members of project teams working from home, and the need to reduce travel expenses due to economic pressures. Even if in-person visits are possible, remote testing can also be more convenient and cost-efficient for all parties involved.

## Methods

We performed a rapid review of studies involving remote usability testing methods for all users and those specifically for older adults and summarized their findings. Rapid reviews are an accepted knowledge synthesis approach that has become popular for understanding the most salient points on emerging or timely topics [6]. Rapid reviews typically do not include an exhaustive set of studies, do not involve formal analyses of study quality, and report findings from prior studies via narrative synthesis [7]. The primary goal of this review was to identify methods for performing remote usability assessments with older adults (if any existed). Secondarily, we wished to summarize the literature on existing remote usability methodologies for any population and existing guidelines on performing in-person usability testing with older adults. Sources for the second goal were largely retrieved while searching for sources to support the primary goal and via a secondary search within Google Scholar.

Our rapid review began with a keyword search on the Google Scholar and Science Direct scholarly databases. This was followed by a supplementary keyword search in top human factors journals and proceedings. Both searches are summarized in Table 1.

**Table 1:** Keywords that were searched for the rapid review.

Search type and sources		Keywords
**Primary search**		
	Google Scholar (database)	*elderly remote usability*, *senior remote usability*, and *older adult remote usability*
	Science Direct (database)	*elderly remote usability*, *senior remote usability*, and *older adult remote usability*
**Secondary search**		
	Ergonomics	*elderly remote usability*, *senior remote usability*, and *older adult remote usability*
	Human Factors	*elderly remote usability*, *senior remote usability*, and *older adult remote usability*
	Applied Ergonomics	*elderly remote usability*, *senior remote usability*, and *older adult remote usability*
	Human Factors and Ergonomics Society Conference Proceedings	*elderly remote usability*, *senior remote usability*, and *older adult remote usability*
	International Journal of Human-Computer Interaction	*elderly remote usability*, *senior remote usability*, and *older adult remote usability*
	International Journal of Human-Computer Studies	*elderly remote usability*, *senior remote usability*, and *older adult remote usability*
	Gerontechnology	*elderly remote usability*, *senior remote usability*, and *older adult remote usability*
	Google Scholar (database)	*usability older adults*, *elderly usability*, *senior usability*, and *remote usability*

We began with Google Scholar to take advantage of its relevance-based sorting feature and broader inclusion of diverse disciplines, academic and practice-based publications, and grey literature [8]. However, we conducted further searches because of the known limitations of Google Scholar, such as its lack of transparency and lack of specialization [9].

In the interest of establishing a starting point for understanding remote usability testing with older adults, we had broad inclusion criteria and did not restrict studies based on their date of publication or an analysis of their quality or peer-review status. We also defined *remote usability* broadly as usability assessments of participants (users) who were in separate locations from the researchers or practitioners. Duplicate studies, as well as studies in which usability was assessed by an expert (eg, heuristic analysis on a website) on behalf of older adults instead of through direct participant feedback, were excluded.

Two authors (JRH and JCB) performed the search in Google Scholar while one author (JCB) performed the search in Science Direct and the human factors sources. Both authors took notes in a shared cloud-based document. We chose a stopping rule based on the assumption that a narrative synthesis of literature is a form of qualitative content analysis [10]. Therefore, we concluded our search when we reached theoretical saturation [11]—a qualitative analysis stopping rule that means that the search continues until results begin to repeat and negligible new categories of information are produced through additional searching.

## Results and Discussion

### Summary of the Search Results

Of all of the sources found, 33 were screened in-depth (18 on remote usability methods and 15 on usability testing with older adults), and 21 were included in this review (16 on various remote usability methods and 5 on usability testing with older adults).

Importantly, sources that provided guidance or information on remote usability testing with older adults (the primary goal of this review) were not found. Therefore, we organized the results according to our secondary goals—summarizing existing methods for remote usability testing and outlining existing guidelines for in-person usability testing with older adults. In this *Results and Discussion* section, we combined the results with our interpretations and discussion to adhere to conventions for narrative reviews. We also present our overall conclusions in the *Conclusions* section.

### Usability-In-Place: The State of the Practice of Remote Usability Testing

Studies on remote usability testing date back to the 1990s [12,13]. Since then, most traditional in-person usability evaluation methods have been attempted remotely. Remote moderated testing has been supported by advances in internet-based software, such as WebEx and NetMeeting, which permit simultaneous video and audio transmissions, screen sharing, and remote control [14,15]. Studies have also used novel methods, such as using virtual reality to simulate laboratory usability testing environments [16] and remotely capturing eye-tracking data [17]. Technologies for unmoderated testing have also evolved, as described elsewhere [18].

Asynchronous methods have long been used to overcome the barriers of time and space. Such methods include conducting self-administered survey questionnaires, using user diaries and incident reports, and obtaining voluntary feedback [19]. Studies have also used activity logging to passively collect use data for analyzing usability [20].

The major remote usability testing methods are described in Table 2 along with key findings from the literature. An important replicated finding was that the results from remote and in-person usability testing were generally similar, although significant differences may have appeared under extenuating circumstances, such as poor product usability or the cognitive difficulty of the usability testing tasks [21].

**Table 2:** Remote usability testing methods and key findings.

Remote usability testing method	Description	Key findings
Synchronous remote testing [14,15,20-23]	In-person testing is simulated by using video and audio transmissions and remote desktop access.	Nearly identical to conventional in-person testing (with comparable results) [14,21-23] Indirect cues and context can be missed [20] Participants can prefer remote testing to in-person testing [22] Management challenges (eg, network issues, remote troubleshooting, and setup) [15,20,22] Users take longer to complete tasks than during in-person testing [15] Users make more errors than during in-person testing [15]
Web-based questionnaires or surveys [14,20,21]	Users fill out web-based questionnaires as they complete tasks or after the completion of tasks.	More time-consuming for users^a^ [14] Less time-consuming for users than lab-based usability testing when usability is poor^a^ [21] Overall usability rated lower when compared to lab-based usability testing [21] Identifies fewer specific usability problems [14] Enables the collection of data from many participants [20] Validity problems with the self-report approach [20]
Postuse interview [24]	Users are interviewed over the phone about the usability of a design (qualitative and quantitative data are collected) after they have completed tasks.	Beneficial for those with disabilities Quantitative data collected are comparable to in-person testing data Qualitative data are less rich compared to in-person testing data In-person testing is better for formative testing; remote testing is better for summative evaluation
User-reported critical incidents or diaries [12,13,19,20]	Users fill out a diary and take notes during a period of use or fill out an incident form when they identify a critical problem with an interface.	Able to capture most high- and moderate-severity incidents^a^ [12,13] Users report fewer low-severity incidents than experts [12,13] Validity problems with self-reports [20] Issues may be underreported compared to those reported via traditional methods^a^ [19]
User-provided feedback [25]	While completing timed tasks, users provide comments or feedback in a separate browser window. Once a task is complete, the user rates the difficulty of the completed task.	The percentage of participants who completed remote testing tasks was the same as the percentage of participants who completed in-person testing tasks No difference in the time taken to complete tasks Able to capture rich qualitative information through typed comments Less observation data captured compared to those captured during in-person testing Captured fewer usability issues in some cases compared to those captured during in-person testing
Log analysis [20]	The actions taken by the user (eg, clicks) are captured for future analysis.	Less intrusive to user Can collect data from many users Unable to capture user intentions or additional context

^a^Conflicting evidence has been found to support both the statement and its opposite in the literature.

The following general benefits of remote usability testing methods were identified:

- Does not require a facility, thereby reducing the time requirements of participants and evaluators and lowering costs [20]
- Can recruit participants from a broader geographic vicinity, thereby allowing evaluators to collect results from a larger and more diverse group of people (including those living in other countries or rural areas or those who are otherwise isolated) [14,23]
- Allows participants to test technologies in a more realistic environment. For example, Petrie et al [24] had people with disabilities perform remote usability testing from the comfort of their own homes. The benefits thereof include the use of a home-based environment that is almost impossible to perfectly replicate in a lab.

Several drawbacks were also described, as follows:

- General agreement that remotely collecting data results in a loss of some of the contextual information and nonverbal cues from participants that are collected during in-person evaluations [15,20,22,24,25]
- Remote usability methods (especially asynchronous methods) appear to result in the identification of fewer usability problems, cause users to make more errors during testing, and are more time-consuming for users [14,15]. However, test participants identified about as many usability issues as those identified by the evaluators, but the participants’ categorization of the identified usability problems were deemed not useful. Contrarily, this was not found by Tullis et al [25] when they compared lab-based usability testing against remote usability testing.
- Dray and Siegel [20] also listed validity problems with self-report methodologies, the inability of log files to distinguish the cause of navigation errors, and management challenges related to troubleshooting network issues and ensuring system compatibility as other drawbacks of remote usability testing. 
- Many of the factors that may affect the validity, reliability, or efficiency of remote usability testing have not been scientifically studied [26]. These include factors such as the characteristics of users (eg, age and literacy), the effect of slow or unstable internet, the type of devices being used, and testing tactics (eg, verbal, printed, or on-screen instructions).

No matter the method, remote usability testing also involves challenges to implementing the methods in natural contexts, namely in home and community settings [27,28]. These challenges include recruiting a representative sample, especially among populations that may be less comfortable with using certain technology, have lower literacy, or are mistrustful of research [26]. McLaughlin and colleagues [26] proposed strategies such as providing access to phone support prior to the start of any web-based testing.

### Remote Usability Testing With Older Adults

Prior work on remote usability testing has been performed with convenience samples of college students [13,14] or healthier and younger adults recruited from workplaces [22,23,25]. We found no published instance of fully remote usability testing with older adults. Diamantidis et al [29] conducted a test of a mobile health system with older individuals with chronic kidney disease. Participants received an in-person tutorial of the system; they used the system at home, received physical materials by mail, and completed a paper diary. Afterward, they returned to complete an in-person satisfaction survey. Petrie et al [24] reported 2 case studies of remote usability testing—one with blind younger adults (n=8) and another with a more heterogeneous group of individuals with disabilities (n=51). They demonstrated the feasibility of remote testing and showed comparable results between in-person and remote testing, although in-person participants in the second study reported more usability problems with the tested website.

Others have described ways to improve in-person usability testing with older adults that may be transferable to remote methods. For example, touch screen devices and hardware that is selected for simplicity may produce better usability testing results with older adults [30-32] and can therefore reduce barriers to remote usability testing. Additionally, the use of large closed captions during a remote testing session has been recommended for older users with visual or hearing impairments. Holden [33] published a Simplified System Usability Scale that was modified for and tested with older adults and those with cognitive disabilities but did not demonstrate its use in remote testing.

**Figure 1:**
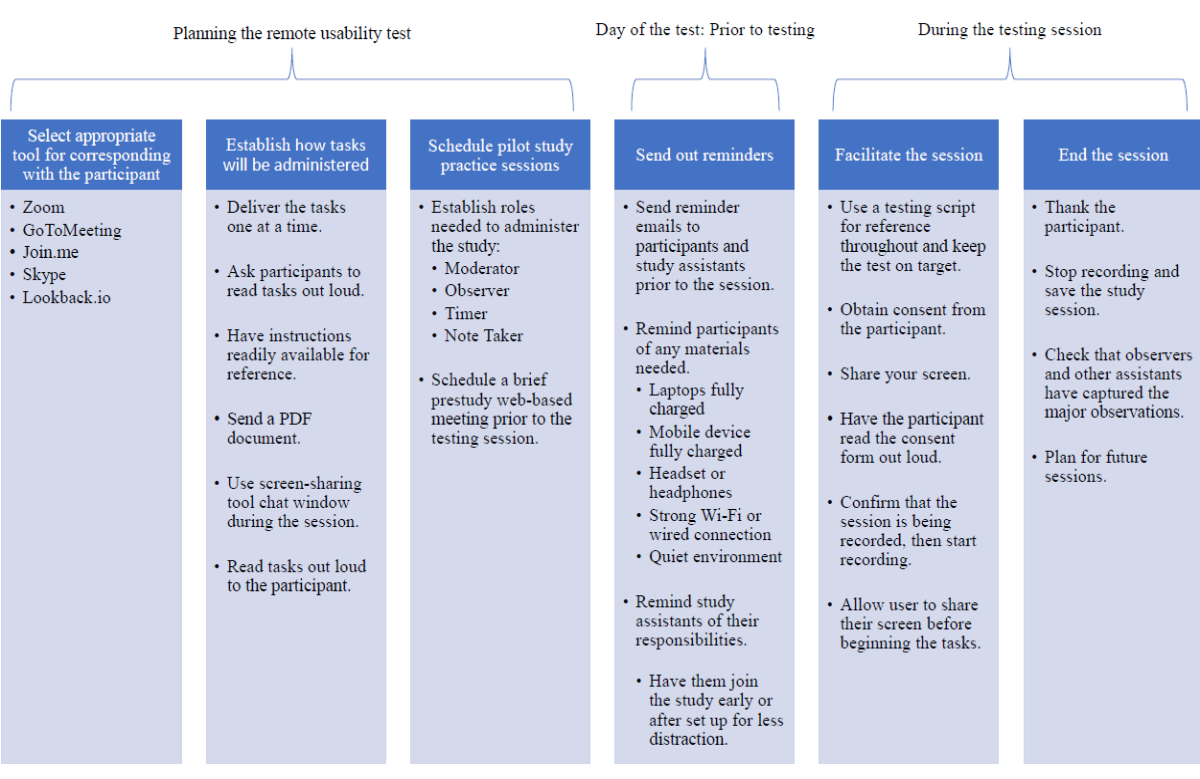
General guidelines for conducting moderated remote usability testing (adapted from the Nielsen Norman Group [34]).

Older adults in remote usability tests may also benefit from non–age-specific strategies for optimizing remote usability testing [34]. These recommendations, which are summarized in Figure 1, include mailing a written copy of instructions, conducting web-based training prior to testing sessions, and sending reminders.

## Conclusions

Our rapid review and synthesis of the literature revealed that remote usability testing still appears to be an emerging field [26] whose great potential is accentuated during major events, such as the COVID-19 pandemic. The decision to pursue the further development of and research on remote usability testing is straightforward, given the apparent advantages, validity, and feasibility of remote usability testing and the need for the method.

The method however must be adapted to and tested with older adults. The use of technology for remote services among older adults in the United States has been increasing [35,36], as has older adults’ proficiency with internet-based technology [37]. A Pew Research Center national survey reported increases in internet use (from 12% to 67%) and the adoption of home broadband (from 0% to 51%) from 2000 to 2016, as well as increases in smartphone (from 11% to 42%) and tablet (from 1% to 32%) ownership from 2011 to 2016 [4]. However, the older adult population is diverse and has different needs compared to those of other groups when it comes to technology and the usability testing of technology. US adults aged 65 years are more likely than their younger counterparts to experience difficulties with physical or cognitive function, including reduced memory capacity, stiff joints or arthritis, and vision or hearing disability [38,39]. These factors and the discomfort with or reduced motivation to use technology elevate the importance of usability testing [40] but ironically may increase the difficulty of conducting remote usability testing. Additional recommendations and best practices will thus be needed to ensure effective and efficient remote usability testing with older adults.

We call on human factors, human-computer interaction, and digital health communities to further develop, describe, and test remote usability testing approaches that will be suitable across diverse populations, including older adults, those with lower literacy or health literacy, and individuals with cognitive or physical disabilities. Progress toward this goal will not only better support homebound or mobility-restricted individuals but may also improve the efficiency, ecological validity, and effectiveness of usability testing in general.
